# The Ocular Surface and How It Can Influence the Outcomes of Keratoprosthesis

**DOI:** 10.1007/s40135-016-0116-x

**Published:** 2016-11-05

**Authors:** Sarah Moussa, Herbert Reitsamer, Josef Ruckhofer, Günther Grabner

**Affiliations:** Department of Ophthalmology and Optometry, Paracelsus Medical University Salzburg, Müllner Hauptstr. 48, 5020 Salzburg, Austria

**Keywords:** Osteo-odonto keratoprosthesis, Stevens-Johnson syndrome, Limbal stem cell deficiency

## Abstract

Severe ocular diseases may result in partial or complete limbal cell deficiency. Besides conservative options, treatment options include conjunctival replacement procedures and limbal autografting. Limbal allografts are an option in patients with bilateral limbal cell deficiency. In many of these cases, a keratoprosthesis (KPro) is the last option to restore functional vision in patients with severe corneal blindness with no other options.

## Introduction

Severe ocular diseases such as Stevens-Johnson syndrome (SJS), mucous membrane pemphigoid (MMP), or severe ocular burns may result in partial or complete limbal cell deficiency. This in turn may lead to severe vision impairment or corneal blindness. Since about 25 years, amniotic membrane has been shown to be a good tool in ocular surface reconstruction—especially in the acute stage. Besides conservative options, like topical and systemic medical therapy as well as contact lenses, chronic stage treatment options include conjunctival replacement procedures and limbal autografting. As an example, the transplantation of limbal stem cells from the healthy fellow eye to diseased limbus has shown excellent results in many cases (autograft). Limbal allografts are an option in patients with bilateral limbal cell deficiency; however, systemic immunosuppression is mandatory in these cases and long-term outcomes might be compromised. In many of these cases, a keratoprosthesis (KPro) is the last option to at least partially restore some useful vision in patients with severe corneal blindness not amenable to conventional corneal and limbal grafting.

Although a large number of KPros have been proposed and tested in small series over the last decades, only two devices, the Boston Type-1 KPro and the osteo-odonto-Kpro (OOKP), have come to the fore. The Boston KPro is completely synthetic, made of PMMA and titanium, and the OOKP is semi-biological (PMMA optric cylinder and bone-dentine-lamina) in concept.

In this review, we briefly highlight the influence of ocular surface diseases such as SJS on the outcome of implanted KPros.

### Ocular Surface Diseases

SJS generally starts with an acute inflammation of the ocular surface followed by chronic conjunctivitis, whereas MMP usually has an insiduous beginning with slow progression of cicatrization leading to cicatricial lid complications with subsequent ocular surface damage and corneal scarring. The resulting destruction of the glands that secrete the tear film leads to a very severe form of dry eye making the management of these autoimmune diseases very difficult. Patients with ongoing conjunctival cicatrizing diseases such as SJS and MMP are extremely challenging and the most difficult to visually rehabilitate. Outcomes of corneal penetrating grafts in such desperate cases are often less than encouraging [1]. Although KPRos offer at least some hope, management is lifelong, complications are frequent, and the outcomes often still disappointing.

### Ocular Burns

A chemical burn usually occurs when a corrosive substance is accidentally introduced into the eye and/or hits periocular tissues [2]. This constitutes a real ophthalmologic emergency requiring immediate evaluation and intensive care. Early findings include corneal and conjunctival epithelial defects, chemosis, conjunctival inflammation, limbal ischemia and edema, corneal stromal haze, sterile ulceration, *edema* , and albeit rarely, even perforation [3, 4].

The most important prognostic factor is the initial degree of the ocular surface damage. Extensive damage to the limbus always leads to at least a certain amount of limbal stem cell deficiency resulting in persistent epithelial defects; these then induce neovascularization and deep corneal scarring [5–8].

Extensive conjunctival burns can also lead to symblepharon, cicatricial entropium or ektropium, and trichiasis [9–11] and can significantly hinder reepithelialization.

If limbal cell transplantation in the form of either *conjunctival-limbal autograft,* [12] *cultivated limbal epithelial transplantation*, [13] *living* - *related concunctival limbal autograft* [14] or *keratolimbal allograft* [15], and *corneal transplantation in its different forms* is not successful, the Boston KPRo remains the first and principal option in unlucky patients where corneal clarity and a normal ocular surface could not be restored with any of the previously mentioned measures [16].

The Boston KPRo study group found an excellent anatomical outcome in patients with chemical burns [17].

The osteo-odonto-keratoprosthesis (OOKP) is considered to be the last resort for patients with bilateral corneal blindness resulting from severe ocular and systemic pathologies including massive chemical injury.

Basically, for all kinds of KPRos, any other surgical alternative available for treatment (e.g., ocular surface reconstruction with stem cell transplantation) should be considered prior to any type of KPRo surgery [18].

### Keratoprosthesis Development and Current Challenges

In 1789, a French ophthalmologist, Guillaume Pellier de Quengsy, first had the idea of replacing an opacified cornea with an artificial cornea. Since then, a number of well-known researchers tried to develop the ideal KPRo using a variety of material and techniques—resulting in often dismal outcomes. In 1905, Zirm implanted the first successful human corneal graft into a human recipient who had suffered from a lye burn with the focus shifting to corneal grafts and the interest in developing newer KPRo models decreased [19].

As the global availability of corneal donor material is limited and with the breakthrough discovery of polymethylmethacrylate (PMMA), as a potentially implantable material in the eye, interest in KPro was growing again [20].

It was realized that for a certain percentage of very severe cases, a corneal transplantation cannot offer a permanent rehabilitation and therefore the interest on KPro is slowly increasing again, with about 150–200 active surgeons worldwide.

The types of KPRos that are currently available vary in design. Most types have their optical cylinder supported with a non-biological skirt that is either hard (e.g., PMMA or titanium in the Boston KPro) or porous (Dacron in the Pintucci-KPro, hydrogel in AlphaCor, or similar Kpros) with the aim of a biological integration of this part of the device.

KPros with *biological* skirts include Strampelli’s OOKP, modified according to Falcinelli (mOOKP), which use an autologous tooth root and alveolar bone or the Tibia-KPro according to Temprano [21–23]. In the last decades, a growing number of KPros have been proposed but only two of them have been implanted in significant numbers and with acceptable documentation: the Boston type-1 KPro (Dohlman, USA) and the mOOKP (Falcinelli, Italy). The Boston KPro is suitable for eyes with a sufficiently wetted surface, whereas the OOKP can also be successful in very dry eyes.

### Osteo-Odonto-Keratoprosthesis (OOKP)

**Fig. 1:**
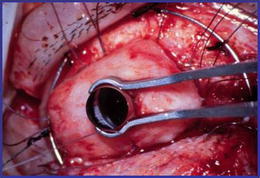
Osteodental lamina and optic

The OOKP was first described by Benedetto Strampelli [21–23] and later modified by Falcinelli [21]. The underlying principle of the OOKP involves the reconstruction of the anterior segment with an osteo-odonto-acrylic/optical cylinder “lamina” and then resurfacing the eye with a biological cover, the buccal mucosal membrane (BMM) which protects the entire complex. A rooted tooth and its alveolar bone are prepared to fashion a plate that is used as carrier for the optical cylinder made of PMMA. This optical cylinder is cemented to the dentine. This implant is called “OOKP lamina” (Fig. 1).

#### Patient Characteristics

Ideal patient characteristics include bilateral corneal blindness resulting from chronic, severe dry eye with limbal stem cell deficiency, such as observed in SJS [22].

The better eye should have no better vision than finger counting at 1 m. Patients who are content with their vision, patients with diagnosed and advanced glaucoma, patients with untreated/or not treatable retinal detachment, and children younger than 17 years have to be excluded [23].

A detailed informed consent discussion is crucial. Cosmesis, the necessity of lifelong follow-ups, several surgeries over a period of about 6 months, and the fact that the recovery of vision is possible only after the last step of the surgery have to be explained carefully to the patient and his family members.

##### Boston Keratoprosthesis

Pioneered by Claes Dohlman, the Boston Kpro was initially made of PMMA with a collar button design consisting of three components: a solid front plate with an optical stem, a back plate with nutritional openings, and a small titanium locking ring. Corneal tissue (the donor corneal button) is “sandwiched” between the two plates. The stem of the front plate is passed through a small (3 mm diameter) aperture of the corneal graft; the back plate is tightly slid over the stem and fixated with the titanium ring. The peripheral corneal skirt is used to suture the complex in the recipient cornea similar to a conventional graft.

The Boston KPro is available in two designs: type I as described is used in sufficiently wetted cases, whereas type II is reserved for severe end stage and very dry ocular surface disease (e.g., SJS, MMP) and requires a permanent tarsorrhaphy through which the anterior part of the optical cylinder protrudes.

### Role of Psychological Counseling

Severe ocular surface diseases like SJS or MMP are chronic processes usually leading to a series of grave complications and multiple treatment challenges both prior and after KPro surgery (e.g., secondary glaucoma, retinal detachment, corneal graft melt, hypotony,…). In addition to a topical and systemic medical and frequent surgical repair, these patients need adequate counseling. Patients should early be referred to either a psychologist or a social worker to assess the patient’s emotional status and coping strategies. The “holistic” approach significantly helps in the management of these difficult conditions [22].

### Clinical Outcomes of Boston Keratoprosthesis and Osteo-Odonto-Keratoprosthesis in Severe Ocular Surface Disease (e.g., in Stevens-Johnsons Syndrome)

A recent WHO study revealed an estimate of nearly 39 million blind and 285 million visually impaired people worldwide. Around 5 million of these cases are due to corneal pathology. In favorable situations (e.g., keratoconus), a corneal transplant has been proven to be highly successful in restoring excellent sight [24]. On the other hand, the clinical outcomes of traditional keratoplasty are far less promising in cases of severe ocular surface disease with deep corneal vascularization, combined with limbal stem cell deficiency, an autoimmune background or a severe chemical injury as all these indications have a great tendency for rapid and severe graft rejection [25].

SJS in its severe form can lead to very severe ocular surface problems such as chronic inflammation with neovascularization, stromal scarring, corneal conjunctivalization, and formation of symblephara. Corneal grafts most of the time fail due to graft rejection and subsequent persistent epithelial defects with danger of stromal ulceration and quite frequent corneal perforation [1].

If limbal cell transplantation (in its different forms of conjunctival-limbal autograft [10], cultivated limbal epithelial transplantation [11], living-related concunctival limbal autograft [12], or keratolimbal [13] and corneal transplantation) is not successful, keratoprosthesis surgery as a last resort can provide long-term clarity of the visual axis.

The keratoprosthesis most frequently implanted worldwide in SJS is the Boston KPro type I. Far fewer patients are treated with Boston KPro type II and OOKP to the best of our knowledge. All these KPros need great surgical experience and a long training, not only for the multiple surgeries required but also for the close and lifelong follow-up that is mandatory. Therefore, only a few centers worldwide provide KPro services.

Initially, the Boston type I keratoprosthesis faired very poorly in SJS (probably the direst outcomes among all indications) with high rates of endophthalmitis and other vision-limiting complications [26].

Over the decades, the advances in both, design and postoperative managements, clinical outcomes improved significantly and the indications for implantation have broadened leading to renewed interest in the use of the Boston KPro in eyes in chronic cicatrizing ocular surface disease [15, 27–29].

In 2008, Dohlman et al. reported outcomes in patients with SJS following six type I and ten type II Boston KPros. They demonstrated a significantly longer preservation of visual acuity than previous reports, with 44 % maintaining a visual acuity >20/70 at the last follow-up (mean follow-up 3.6 ± 1.5 years; range 10.2 months–5.6 years) with no significant difference in visual acuity or retention rate between both keratoprosthesis designs [30].

Although in the last years many studies with Boston KPros included a few select patients with SJS, none have specifically focused on the outcomes of the Boston type I KPro in SJS. Recently, Aldave et al. in their multicenter, retrospective comparative study compared the outcomes of Boston KPro I in SJS and non-SJS patients [26]. They reported a significantly better outcome in visual acuity in the SJS group. They postulated this might be due to the significantly lower rate of preoperative glaucoma in their SJS series—as the diagnosis of preoperative glaucoma had been reported to be a risk factor for loss of >20/200 CDVA after keratoplasty surgery [26]. In addition, it has been shown in an earlier report that eyes with severe ocular surface disease have the lowest prevalence for glaucoma [31]. On the other hand, Yaghouti et al. had reported in their study that none of the eyes with SJS retained 20/200 vision or more at the 5 years follow-up after KPro implantation [32].

**Fig. 2:**
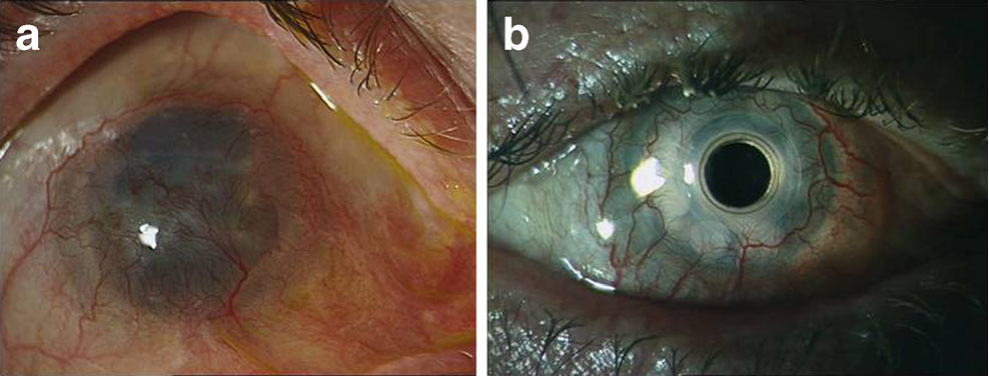
**a** Preoperative clinical photograph of the right eye of a patient postlye burn. Corneal scarring showing deep vascularization in all four quadrants. Patient had two times a penetrating keratoplasty. Vision: hand movement. **b** Postoperative clinical photograph of the patient 18 months after Boston KPro implantation. Vision: 0.8

Retention rates of Boston KPro type I were reported to range between 74 and 100 % at the last follow-up (from 1 week to 85 months) [33] Fig.Fig. 2a, b. The outcomes seemed to be dependent on the primary indication. Autoimmune diseases, chemical injury, and deep corneal vascularization were found to be associated with lower retention rates [27, 32–37]. Aldave et al. also stated that in patients with SJS, the failure rate was found to be 4.5 times higher than that for other indications. Over three quarters of these losses resulted from sterile corneal stromal necrosis, which has been documented to occur more frequently in eyes with chronic conjunctival inflammation [26, 27, 32, 38].

They also could show that patients with SJS were developing significantly more often a microbial keratitis than the group without SJS [26]. This higher incidence might be due to the increased incidence of persistent epithelial defects in SJS eyes, leading to a significantly higher risk of infectious keratitis [39]. The higher risk of fungal keratitis in Boston Kpro patients with SJS could be correlated with bandage lens wearing and the continuous topical application of vancomycin [27].

Another grave complication in this setting is the ever-present risk of developing an endophthalmitis. Two studies have suspected this increased risk to be due to the underlying systemic inflammatory disease [40••, 41]. In contrast, others state that there is no higher risk claiming even a reduced incidence in these cases. In contrast to the conflicting reports with endophthalmitis, there seems to be no higher risk for retroprosthetic membrane, elevated intraocular pressure, sterile vitritis, or cystoid macular edema in Boston KPro patients with SJS [26, 41].

The OOKP surgical procedure as described in detail in the “Vienna-Rome protocol” has been practiced in the last four decades with minor modifications in technique in only a handfull number of centers worldwide [16, 20].

In a systematic review of surgical outcomes and complication rates, Tan et al. included eight case series with OOKP from 1950 to 2010. They stated that the most common indications are SJS and burns (both thermal and chemical). Three studies described a retention rate of 81 % (range 46–98 %) at 20 years. Severe sight-threatening, intraoperative complications, such as suprachoroidal hemorrhage, occurred in 3–5 % of eyes in all eight studies. Vitreous hemorrhage was the most common intraoperative complication (range 0–52 %). The most dangerous (e.g., blinding in the long-term follow-up) and difficult to treat sight-threatening complication was glaucoma (range 7–47 %). Other serious complications included endophthalmitis (range 2.0–8.3 %), resorption of the lamina (range 2.0–43.0 %), and retinal detachment (2.0–26 %). Across all the eight studies, 52 % (range 46–72 %) of patients achieved vision of more than 6/18 [42••].

Liu et al. included 36 patients in their analysis. Their most frequent preoperative diagnoses also were Stevens-Johnsons syndrome (*n* = 16, or 44 %), severe thermal or chemical burns (*n* = 6, or 17 %), and mucous membrane pemphigoid (*n* = 5, or 14 %). They reported a retention rate of 72 % and an improvement in visual acuity in 83 %. Of these 36 patients, 78 % achieved 6/12 or better with a mean follow-up of 3.9 years (range 6 months–9 years). The main factor resulting in final anatomical failure was resorption of the OOKP lamina, which occurred in 19 % [23]. However, none of the abovementioned case series differentiated SJS patients from other indications and the long-term outcomes for a SJS series only have not been reported yet.

Another important complication especially occurring in SJS is necrosis of the mucous membrane covering the lamina. Across various studies, a prevalence ranging from 8 to 48 % was observed [42••]. This severe complication is mainly due to inadequate vascularization of the buccal mucosal membrane on the ocular surface. Minor defects can be treated conservatively with increased lubrication or a scleral shield. Persistent defects should be treated with a range of oculoplastic procedures like rotational flaps and free mucosal grafts [43]. Iyer et al. described mucosal necrosis after OOKP in quite some detail and showed that this disastrous complication had a specially high prevalence in SJS (10 of 24 eyes, 41.7 %) as compared to non-SJS eyes (3 of 26 eyes, 11.5 %) [44]. This is similar to the finding of Basu et al.—they reported a prevalence of mucosal necrosis in 15 of 30 eyes (50 %) [45]. Liu et al. and Tan et al., who had comparable proportion of SJS patients in their own series, reported similar rates of necrosis [23, 42••]. These high prevalences might be a consequence of some subclinically ongoing changes in the transplanted oral mucosa following an acute attack of SJS that leads to ischemic necrosis or might result following minimal localized trauma and exposure with desiccation [44]. The structure, with a sufficient thickness in all parts anterior to the lamina, and a good vascularization of the mucosal graft are all critical. It is important to mention that even minor mucosal necrosis can progress to rapid lamina resorption and endophthalmitis [46]. This makes a very close follow-up of these SJS patients with functioning OOKP mandatory.

## Conclusion

OOKP is a time-tested procedure (with up to 38 years of follow-up in single cases) and can therefore be considered the “gold standard” with which all other KPros have to be compared, especially in severe ocular surface diseases with extreme dryness and surface keratinization.

However, it comes with a high price: multiple surgeries, oral morbidity (albeit a minor discomfort in most cases), a host of partially very severe, sight-threatening complications, an ungainly appearance of the anterior segment of the eye, and frequent hospital visits place a high burden on the patient and the relatives.

The Boston KPro can easily be repeated in case of implant failure and is surgically far less challenging, carrying a lower chance of long-term restoration in these cases, however.

For both OOKP and Boston KPro, the level of available social care and self care has to be clearly evaluated well before offering a keratoprosthesis. Selection of the correct device for the adequate patient and thorough counselling in consideration of his diagnosis are crucial for optimizing outcomes. Any kind of keratoprosthesis requires a lifelong patient follow-up!

Boston KPro can be considered the device of choice for a sufficiently moist, well-blinking eye, while OOKP is the gold standard for eyes with severe ocular dryness and lacking good lid function. Keratoprostheses have enjoyed quite high success rates, especially in non-autoimmune disorders.

However, in patients with severe ocular diseases of suspected autoimmune pathogenesis continued innovations and controlled clinical trials are direly needed.
